# Evaluation of the Feasibility of NaCaPO_4_-Blended Zirconia as a New CAD/CAM Material for Dental Restoration

**DOI:** 10.3390/ma14143819

**Published:** 2021-07-08

**Authors:** Ting-Hsun Lan, Yu-Feng Chen, Yen-Yun Wang, Mitch M. C. Chou

**Affiliations:** 1 Department of Dentistry, Division of Prosthodontics, Kaohsiung Medical University Hospital, Kaohsiung 807377, Taiwan; 2 School of Dentistry, College of Dental Medicine, Kaohsiung Medical University, Kaohsiung 807378, Taiwan; wyy@kmu.edu.tw; 3 Department of Dentistry, Division of Oral and Maxillofacial Surgery, Kaohsiung Medical University Hospital, Kaohsiung 807377, Taiwan; alpha0718@yahoo.com.tw; 4 Center for Cancer Research, Kaohsiung Medical University, Kaohsiung 807378, Taiwan; 5 Translational Research Center, Kaohsiung Medical University Hospital, Kaohsiung 807377, Taiwan; 6 Department of Medical Research, Kaohsiung Medical University Hospital, Kaohsiung 807377, Taiwan; 7 Department of Materials and Optoelectronic Science, National Sun Yat-sen University, Kaohsiung 804201, Taiwan; mitch@faculty.nsysu.edu.tw

**Keywords:** zirconia, NaCaPO_4_, CAD/CAM material, milling property

## Abstract

The computer-aided design/computer-aided manufacturing (CAD/CAM) fabrication technique has become one of the hottest topics in the dental field. This technology can be applied to fixed partial dentures, removable dentures, and implant prostheses. This study aimed to evaluate the feasibility of NaCaPO_4_-blended zirconia as a new CAD/CAM material. Eleven different proportional samples of zirconia and NaCaPO_4_ (*x*Z*y*N) were prepared and characterized by X-ray diffractometry (XRD) and Vickers microhardness, and the milling property of these new samples was tested via a digital optical microscope. After calcination at 950 °C for 4 h, XRD results showed that the intensity of tetragonal ZrO_2_ gradually decreased with an increase in the content of NaCaPO_4_. Furthermore, with the increase in NaCaPO_4_ content, the sintering became more obvious, which improved the densification of the sintered body and reduced its porosity. Specimens went through milling by a computer numerical control (CNC) machine, and the marginal integrity revealed that being sintered at 1350 °C was better than being sintered at 950 °C. Moreover, 7Z3N showed better marginal fit than that of 6Z4N among thirty-six samples when sintered at 1350 °C (*p* < 0.05). The milling test results revealed that 7Z3N could be a new CAD/CAM material for dental restoration use in the future.

## 1. Introduction

As a promising prosthodontic material, zirconia exhibits excellent biocompatibility while possessing outstanding mechanical properties, such as mechanical strength, wear resistance, and thermal stability. It also has a unique phenomenon known as “transformation toughening”, which helps to halt crack propagation [1]. A zirconia crown has several advantages, such as high flexural strength (1020 MPa) and a low tendency to fracture (~9 MPa⋅m^−^^1/2^) [2]; however, it also has the disadvantage of excessive hardness (~1300 Hv) compared to enamel, which leads to an abrading effect on the opposing natural tooth if its surface is not fully polished [3].

Hydroxyapatite (HA) is a form of calcium phosphate, which is a desirable biomaterial for use. Due to its excellent biocompatibility, HA has been classified as a promising material in many biological applications [4]; however, the low mechanical strength and intrinsic brittleness of HA make it unsuitable as a load-bearing implant fixture and limit its use as a bioactive layer on metal/plastic porous implant materials [5]. 

Kong et al. [6] confirmed that adding some inclusions to ZrO_2_, such as mullite (ZrO_2_-toughened mullite) and alumina (ZrO_2_-toughened alumina), can improve its mechanical properties. Casellas et al. [7] demonstrated that the addition of zirconia particles enhanced the fracture toughness, as exhibited by the alumina matrix. Towler et al. [8] pointed out that the use of ZrO_2_ inclusions can also improve the mechanical properties of HA. Generally, a higher sintering temperature is required to make ZrO_2_ completely dense. Under high-temperature sintering, dense HA–ZrO_2_ composite production might be problematic and cause HA to decompose into fragile or absorbable calcium phosphate forms, which also limits the application of HA as a biomedical material in the body [5].

Some researchers have recently reported that orthorhombic-NaCaPO_4_ (sodium-calcium phosphate) crystalline phases were detected while the HA sample was calcined at 600~1150 °C [9,10,11]. Suchanek et al. pointed out that the β-NaCaPO_4_ interphase acts as a path for crack deflection and debonding in the laminate, which indicates that this material can work as a weak interphase in HA ceramics. Most importantly, they found that β-NaCaPO_4_ also exhibits high biocompatibility and bioactivity [12]. In addition, Kangasniemi et al. [13] pointed out that after immersing the β-NaCaPO_4_/active glass-sintered composite body in simulated body fluid for a while, the β-NaCaPO_4_-containing composites had a more substantial effect than did HA for the formation of the Ca–P apatite layer on the sintered body surface.

Most research on NaCaPO_4_ has focused on its luminescent properties [14,15], while its application in biomedicine has primarily been on orthopedic materials [16]. Therefore, little research on its applicability to dental materials has been published. One of the purposes of this study is to incorporate NaCaPO_4_ into ZrO_2_ and observe the effect of different contents of NaCaPO_4_ on the mechanical properties of ZrO_2_ while evaluating the application potential of NaCaPO_4_-blended zirconia as a computer-aided design/computer-aided manufacturing (CAD/CAM) material for dental restorations. In addition, performing indirect restorations of minor decay was a suitable way to test the milling property of CAD/CAM materials. Therefore, the other purpose of this study is to preliminarily evaluate the milling property of CAD/CAM materials by measuring the marginal discrepancy of NaCaPO_4_-blended zirconia inlays.

## 2. Materials and Methods

### 2.1. Sample Preparation

**Figure 1:**
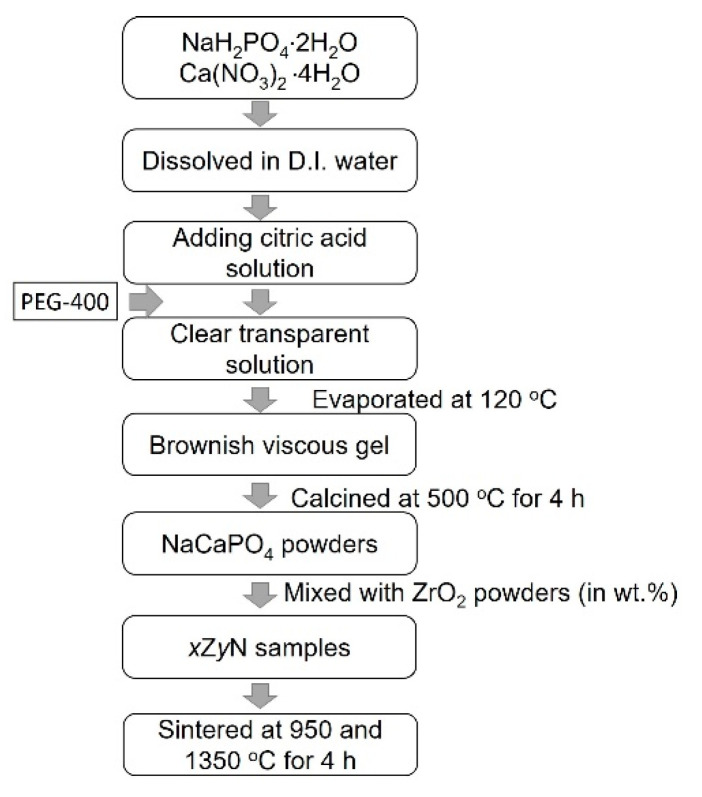
Flow chart of sample preparation procedures conducted in this study.

A sol–gel process was used to prepare NaCaPO_4_. High-purity samples of sodium dihydrogen phosphate (NaH_2_PO_4_·2H_2_O, reagent grade, supplied by NIHON SHIYAKU REAGENT, Kyoto, Japan) and calcium nitrate tetrahydrate (Ca(NO_3_)_2_·4H_2_O, reagent grade, supplied by NIHON SHIYAKU REAGENT, Kyoto, Japan) were used as starting materials, citric acid (C_6_H_8_O_7_, reagent grade, supplied by NIHON SHIYAKU REAGENT, Kyoto, Japan) was used as a chelating agent, and PEG 400 (NIHON SHIYAKU REAGENT, Kyoto, Japan) was added to the mixture as a dispersing agent. Initially, stoichiometric amounts of 15.601 g of NaH_2_PO_4_·2H_2_O, 23.615 g of Ca(NO_3_)_2_·4H_2_O, and 84.06 g of citric acid were dissolved in 600 mL of deionized water and stirred until entirely dissolved; then, 3.6 g of PEG-400 was added to the solution while stirring continued. A transparent solution was obtained, which was then evaporated slowly at 120 °C until a viscous and thick brown gel was formed. Subsequently, the gel was calcined at 500 °C for 4 h, and a white NaCaPO_4_ powder was obtained. The preparation procedure of NaCaPO_4_ is presented in Figure 1. Afterwards, the calcined NaCaPO_4_ powder was ground with an agate mortar and pestle and mixed with a commercial nano-sized ZrO_2_ powder (TZ-3Y-E, Tosoh Corp., Kyoto, Japan) at different weight ratios (hereafter, *x*Z*y*N denotes the sample with different weight ratios *x*:*y* of ZrO_2_ and NaCaPO_4_). The disk-shaped specimens were processed by the cold isostatic pressing method into a cylindrical rod that was 15 mm in diameter and 10 mm in length. After the peeling of the rod surface, all specimens were sintered in a SiC furnace with a heating rate of 5 °C/min and maintained at 950 °C and 1350 °C for 4 h, respectively. After sintering, the specimens were cooled in the furnace. It is worth noting that samples with various weight ratios might cause different results depending on the heating profiles, but the influence of heating profiles is not the focus of this study; hence, all *x*Z*y*N samples were sintered under the same conditions.

### 2.2. Sample Characteristics

The phase identification of the sintered ZrO_2_-NaCaPO_4_ specimens was determined by X-ray diffraction (XRD, Advanced D8, Bruker Corp, Billerica, MA, USA) with monochromatic Cu Kα radiation (λ = 0.1540 nm) and a Ni filter. The operation’s current and voltage were 20 mA and 30 kV, respectively, at a scanning rate (2θ) of 2°/min. The phase content of the tetragonal phase was calculated with the following equation [17]:

(1) $$f_{T}=\frac{I_{T}\left(111\right)}{I_{T}\left(111\right)+I_{M}\left(\bar{1}11\right)+I_{M}\left(111\right)}$$

where *f*_T_ denotes the fraction of the tetragonal ZrO_2_ phases; *I*_T_ (111) is the intensity of the tetragonal ZrO_2_ (111) reflection; and *I*_M_ ($\bar{1}$11) and *I*_M_ (111) are the intensities of the monoclinic ZrO_2_ ($\bar{1}$11) and (111) reflections, respectively.

The linear shrinkage of samples during the sintering process was determined by the following Equation (2):

Shrinkage = [(*D*_g_ − *D*_s_)/*D*_g_] × 100% (2)

where *D*_g_ and *D*_s_ are the diameters of the green sample and sintered samples, respectively. The open porosities of the sintered bodies were measured using an immersion method. Sintered bodies were weighed in air and then placed under a high vacuum for 1 h. Water was then introduced into the vacuum system, and samples were kept under this condition for 2 h and then removed from the vacuum, after which redundant water from the surface was allowed to be absorbed. Finally, the samples were weighed, first in air and subsequently in water. 

Vickers indentation tests were executed using a diamond indentation technique (Leco M-400-G1 Hardness Tester, Leco, MI, USA). The samples sintered at 1350 °C were indented with a 4.9 N load for a dwell time of 15 s. Each sample was measured at eight different positions to calculate the average value. The resultant indents were measured and used to calculate hardness using the following formula:

Hv = (1.88544 × *P*)/*d*^2^ (3)

where Hv is the Vickers hardness, *P* the applied load, and *d* is the mean of the measured indenter diagonals.

**Figure 2:**
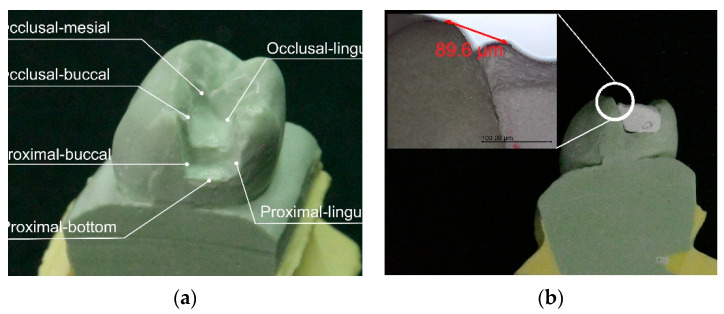
(**a**) Schematic diagram for measuring the marginal discrepancy of the inlays in this study is shown. (**b**) The marginal discrepancy of occlusal–buccal to sample is shown.

Each specimen was milled from the center of the block, with the experimental block fixed in place using a resin block with a diameter of 20 mm to fit the milling machine. All samples were milled by the same open CNC system milling center (Ardenta CNC mill, CS100, Tainan, Taiwan) with new tungsten carbide burs used during the milling process, while the milling properties were recorded by a milling tool (DT100-5A, ARIX CNC MA-CHINES CO., Tainan, Taiwan). The specimens were then pushed by the milling tool under rotation at 40,000 rpm with a constant force of 0.98 N. The specimens were all milled into the same class II inlays and placed into the same mandibular left first molar cavity. The marginal integrity of the specimens and the marginal discrepancy were measured with image analysis software under a digital optical microscope (OM, Olympus BX51, OLYMPUS, Tokyo, Japan), with OM evaluating the marginal fit of the inlays from six selected locations (as shown in Figure 2a, and the schematic for measurement of the marginal discrepancy of occlusal–buccal is shown in Figure 2b. G power analysis was used to estimate the required sample size, assuming two test groups, an effect size of 0.98, a 0.05 probability of a type I error, and power of 0.80. The sample size was thus determined to be 18 per group.

### 2.3. Statistical Methods

The mean and standard deviation among the different blending ratios and the marginal discrepancy were calculated with the Kruskal–Wallis test followed by Dunn’s multiple comparison test. The deviation of data gained from this study of 7Z3N and 6Z4N was analyzed by using the two-sample *t*-test. All statistical analyses were performed using SPSS software (version 20.0 for windows, SPSS, v20; IBM Corp, Armonk, NY, USA). A statistical significance value of *p* < 0.05 was used in all tests. 

## 3. Results

**Figure 3:**
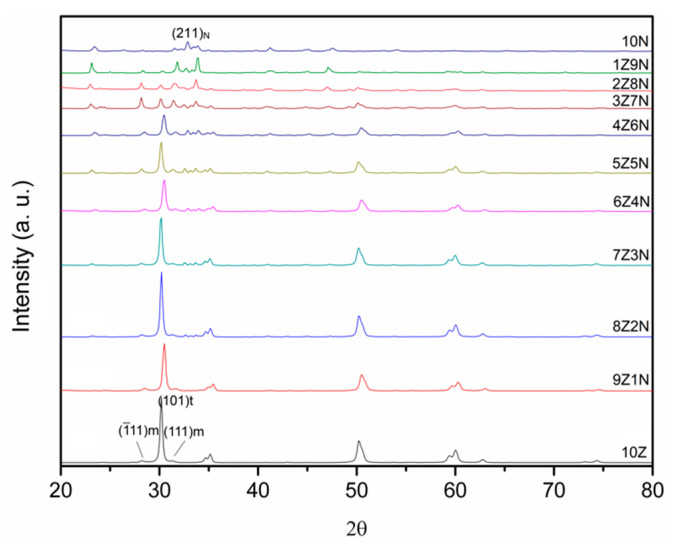
XRD patterns of ZrO_2_ mixed with NaCaPO_4_ at different weight ratios.

Figure 3 shows the XRD patterns of ZrO_2_ mixed with NaCaPO_4_ at different weight ratios after calcination at 950 °C for 4 h. It can be seen that tetragonal ZrO_2_ (JCPDS no. 88-1007) is the major phase accompanied by the ($\bar{1}$11) and (111) reflection peaks of monoclinic ZrO_2_ (JCPDS no. 88-2390) in sample 10Z. In contrast, sample 10N reveals the reflection peaks of NaCaPO_4_ (JCPDS no. 23-1193).

**Figure 4:**
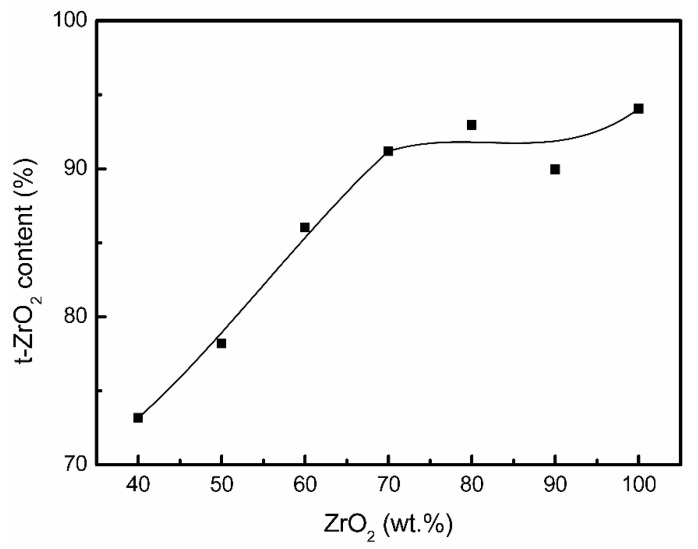
t-ZrO_2_ phase content with different ZrO_2_ weight ratios.

With the increase in the blended NaCaPO_4_ content, samples 9Z1N and 8Z2N still show similar XRD patterns to that of sample 10Z; however, the intensity of the (101) reflection peak of t-ZrO_2_ gradually decreased, and the weak NaCaPO_4_ (120) reflection peak first appeared in sample 8Z2N. Secondly, based on the XRD patterns of sample 7Z3N to sample 3Z7N, it can clearly be seen that t-ZrO_2_, m-ZrO_2_, and NaCaPO_4_ coexist. Additionally, this reveals that both the quantity and intensity of reflection peaks of NaCaPO_4_ increase and that the intensity of the (101) reflection peak of t-ZrO_2_ continues decreasing. Interestingly, the relative intensity of the ($\bar{1}$11) and (111) reflection peaks of m-ZrO_2_ becomes continuously stronger. This phenomenon might suggest that NaCaPO_4_ inhibits the formation of the t-ZrO_2_ phase during the calcination process. Finally, the XRD patterns of samples 3Z7N to 10N almost show the major phase of the NaCaPO_4_, where the peak intensity of ZrO_2_ is very weak or even non-existent. Moreover, according to the XRD result and Equation (1) (the calculated t-ZrO_2_ phase content is shown in Figure 4), the curve reveals that the tetragonal phase content is almost maintained above 90% from samples 10Z to 7Z3N but linearly decreases from samples 7Z3N to 4Z6N. 

**Figure 5:**
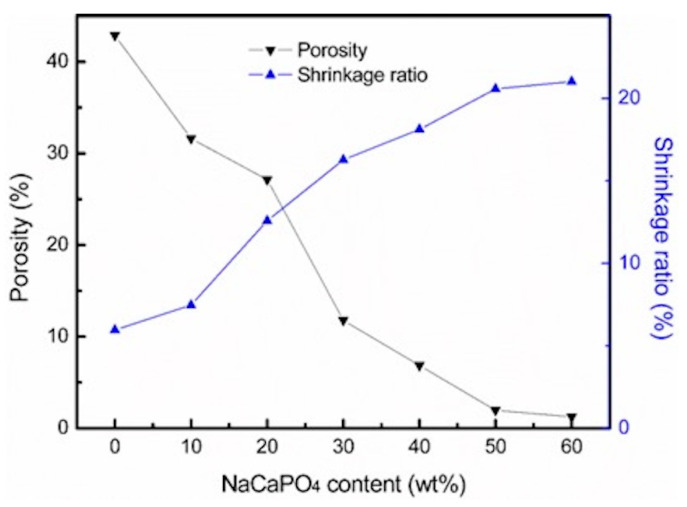
Porosity and volume shrinkage ratio of samples at different NaCaPO_4_ weight ratios.

**Figure 6:**
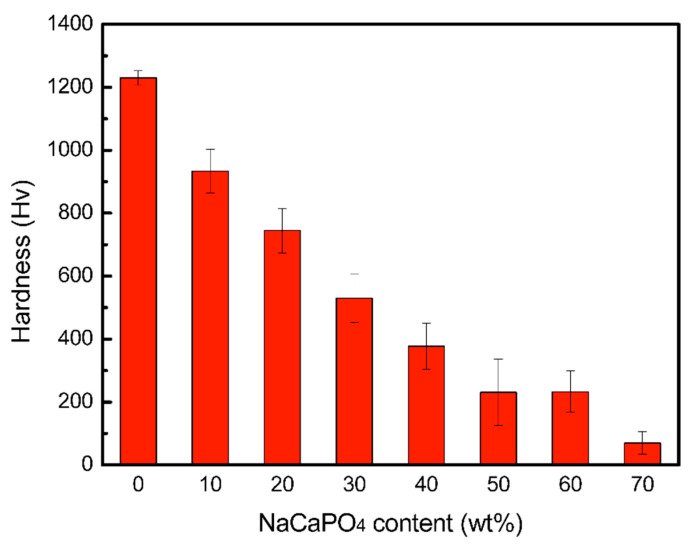
The hardness of samples at different NaCaPO_4_ weight ratios.

Figure 5 shows both the porosity and shrinkage ratio of the ZrO_2_-NaCaPO_4_ specimens sintered at 1350 °C. The porosity decreases from 42.9% to 1.2% and the shrinkage ratio increases from 6.0% to 21.0%, with the NaCaPO_4_ weight ratio increasing from 0% to 60%. The results indicate that NaCaPO_4_ could promote the densification of the sintered body, which could be attributed to the liquid phase sintering, with NaCaPO_4_ playing the role of flux in the sintering process. Figure 6 reveals the hardness of samples 10Z to 3Z7N; when the content of NaCaPO_4_ increases, the hardness value gradually decreases from the highest value of 1230.0 ± 22.5 Hv for sample 10Z to 69.8 ± 35.8 Hv for sample 3Z7N. This means that adding NaCaPO_4_ to ZrO_2_ could reduce the mechanical strength of ZrO_2_, which has a similar effect as adding HA. The hardness of tooth enamel is approximately 283–374 Hv [18], which is close to the hardness obtained by samples 7Z3N and 6Z4N, so only they were selected to evaluate the milling property in this study. These two specimens were successfully milled into two inlays that would fit in the inlay cavity on the gypsum dental model.

**Figure 7:**
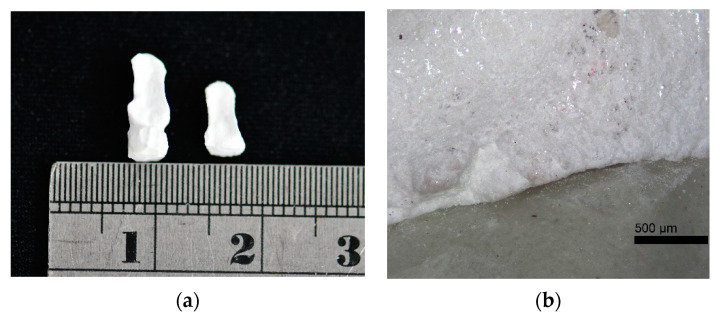
(**a**) OM image of samples sintered at 950 °C (right) and 1350 °C (left), showing the better marginal integrity at 1350 °C. (**b**) Marginal discrepancy of sample 7Z3N sintered at 1350 °C.

The OM images used to measure the marginal gap of samples 7Z3N and 6Z4N that were sintered at 950 °C and 1350 °C for 4 h, respectively, are shown in Figure 7, with the marginal integrity revealing that sintering at 1350 °C is better than that at 950 °C (Figure 7a). The marginal discrepancy of samples 7Z3N and 6Z4N sintered at 1350 °C for 4 h are also observed, revealing that sample 6Z4N is slightly damaged on the occlusal edge. Compared with sample 7Z3N, sample 6Z4N has a more significant discrepancy over the distal marginal area (Figure 7b). 

The edge damage results of samples 7Z3N and 6Z4N after cutting are shown in Table 1. The measurement result of sample 7Z3N was 0.90 ± 0.96 mm, and the measurement result of sample 6Z4N was 7.16 ± 10.65 mm, which shows that the deviation of sample 6Z4N after milling was very extensive and, according to the two-sample *t*-test, shows a significant difference between these two samples (*p* < 0.001). Due to sample 7Z3N having better results than those of sample 6Z4N, the authors further analyzed the marginal discrepancy of sample 7Z3N at six different margins (as shown in Figure 2), with the statistical results listed in Table 2. The difference between groups of 7Z3N is *p* > 0.05, meaning that there is no marginal discrepancy at either position. 

## 4. Discussion

According to the XRD results, when the weight ratio of NaCaPO_4_ is more than 70%, it is almost impossible to detect the presence of ZrO_2_, and although the sintered body tends to become denser by adding NaCaPO_4_, this reduces the hardness of the composite body and the relative phase content of t-ZrO_2_. Ghannam et al. pointed out that Ca, P, and Na atoms prefer to migrate to the grain boundary due to the low melting point of β-NaCaPO_4_ [19]. It is speculated that these atoms on the grain boundaries form a liquid phase at the sintering stage, filling the pores to promote densification and making the sintering phenomenon more obvious. This result is also similar to Evis’s report [20], where it was found that when the HA in the sintered composite material decomposes into water, the porosity of the sintered composite material increases with an increased concentration of zirconia in the composite material.

For the phase transformation mechanism from t- to m-ZrO_2_, many of these are based on a reaction with water on the crystal surface. To date, the presence of metastable tetragonal zirconia at room temperature is due to the stabilization of oxygen vacancies in the crystal lattice, where the phase change of pure tetragonal zirconia can be initiated by reacting with water [21]. In this study, because of the high-temperature liquid phase sintering, as the liquid phase fills the pores, the contact area of the zirconia surface with water increases, which in turn induces the phase transformation. 

However, it is known that phosphates can stabilize the tetragonal phase in zirconia. Skovgaard et al. [22] proposed that this mechanism is caused by anionic interactions with phosphate ions on the zirconia surface, and that the tetragonal phase stabilization is a result of the hindered reaction of water on the surface. In the case of NaCaPO_4_, which belongs to the phosphate group, our results did not follow those of Skovgaard et al.; therefore, it is speculated that the migration phenomenon of Ca^2+^ and Na^+^ is more evident than that of PO_4_^3−^, which would explain why the above situation did not occur.

Curran et al. [5] mixed ZrO_2_ with HA, observed the effect of different sintering temperatures and methods on the material properties of the mixture, and indicated that as the content of ZrO_2_ increases (0~5 wt.%), the relative density and porosity will be slightly reduced. That result is partially consistent with this study, indicating that an increase in ZrO_2_ will increase the porosity. Although the highly interconnected open porosity is believed to promote osseointegration, its effect on dental prostheses is still unknown. Additionally, the interconnected open porosity increased with an increase in the ZrO_2_ loadings while slightly affecting the mechanical properties, whereas the hardness of the composites decreased with ZrO_2_ content, resulting from increased ZrO_2_ loadings retarding mechanical strength due to an increased formation of TCP and increasing porosity. This is slightly different from our results, and the reason might be due to the difference in the mixing ratio of ZrO_2_. A small amount of ZrO_2_ may have little effect on the properties of HA-ZrO_2_, but when the mixing ratio is increased, it will observably change the material properties.

Dentists and scientists have raised concerns over increased wear and/or damage to enamel by zirconia when it is used without veneering. It is currently believed that well-polished zirconia does not cause excessive wear or damage to opposing dental prostheses, thereby resulting in less antagonistic wear than other ceramics [23,24]. Moreover, Stober et al. [24] proposed that monolithic zirconia crowns generated more wear on opposing enamel than did natural teeth wear to enamel and that, accordingly, an innovative, well-polished, and lower hardness crown needs to be developed. It was found that the 7Z3N and 6Z4N in this study could reduce the hardness of zirconium dioxide. Further research should be carried out to apply these modifications to dental restoration materials, which is expected to reduce damage to the opposing enamel.

Marginal fit is essential for the longevity of restoration, but there is still an undetermined threshold value. Gemalmaz and Kukrer [25] evaluated the marginal gap and discrepancy of indirect class II restoration via in vivo and in vitro conditions. The gap showed the shortest distance, and the discrepancy exhibited the furthest margin. The gap in vivo mean values for occlusal and proximal locations were recorded as 73 μm and 132 μm, respectively, while the maximum marginal discrepancy in vitro reached occlusal and proximal locations recorded as 240 μm and 350 μm, respectively. Four CAD/CAM block materials (Cerasmart, IPS e.max CAD, Vita Enamic, and Lava Ultimate) were used to measure the marginal discrepancy ranging from 119 ± 55 μm to 234 ± 51 μm [26]. Additionally, a systematic review showed that for ceramic inlays and onlays, the marginal gap varied between 23 μm and 230 μm [27], while Hamza and Sherif [28] evaluated the marginal gap of five different monolithic zirconia restorations milled with different CAD/CAM systems, and their marginal gap ranged from 39.3 ± 2.3 μm to 22.8 ± 8.9 μm. 

Although the measurement method and position of previous studies are not the same, the marginal discrepancy of the 7Z3N in this study is 0.90 ± 0.96 mm, being somewhat unstable compared to that in the literature, and this could be attributed to different materials with different milling parameters. The prototype of the laboratory disk still requires further enhancement. Further research could focus on other milling parameters for obtaining a better marginal integrity and marginal fit with more sample sizes. In addition, the marginal discrepancy at different positions is not statistically different in 7Z3N, which means that the marginal discrepancy is somewhat consistent on all sides. For the new composite material NaCaPO_4_:ZrO_2_, there are still many issues worthy of future in-depth discussion. Even so, from the perspective of its material properties and milling results, this study preliminarily evaluated the potential of 7Z3N as a dental prosthetic material.

This study contained a few limitations. First, as this study intended to compare the changes in sample properties between different ratios, the same sintering conditions were used, and, as such, only the influence of sintering profiles on material properties was investigated. Second, given the slightly insufficient sample size of this experiment, the results of this comparison with the marginal discrepancy of other commercial products is not very stable. Finally, other milling properties, such as contact with the cavity surface, the luting cement effect, and different milling parameters, are not discussed herein and remain to be examined in future studies.

## 5. Conclusions

ZrO_2_ mixed with NaCaPO_4_ at different ratios is herein successfully prepared and discussed, and an evaluation of the feasibility of a NaCaPO_4_-ZrO_2_ composite material is also presented. The phase evolution of each *x*Z*y*N sample is obtained from the XRD result, showing that the content of t-ZrO_2_ gradually decreases with an increase in the content of NaCaPO_4_. Moreover, accompanied by the increase in NaCaPO_4_ content, the liquid phase sintering becomes more prominent, which improves the densification of the sintered body and reduces its porosity. Blending NaCaPO_4_ with ZrO_2_ can effectively reduce the hardness of ZrO_2_, and the hardness obtained by samples 7Z3N and 6Z4N is close to that of tooth enamel. The milling test results reveal that 7Z3N could become a new CAD/CAM material for dental restoration use in the future.

## Figures and Tables

**Table 1:** Marginal discrepancy of samples 7Z3N and 6Z4N sintered at 1350 °C for 4 h (N = 36).

Sample	Mean ± SD (mm)	*p*-Value *
7Z3N	0.90 ± 0.96	*p* < 0.001
6Z4N	7.16 ± 10.65	

* Two-sample *t*-Test (*p* < 0.05).

**Table 2:** Statistical results of marginal discrepancy of sample 7Z3N (N = 18).

Position	Mean ± SD (mm)	*p*-Value * (MC)
Occlusal–lingual	0.74 ± 0.44	*p* = 0.067
Occlusal–buccal	1.41 ± 1.55	
Occlusal–mesial	1.10 ± 0.98	
Proximal–bottom	0.93 ± 1.01	
Proximal–lingual	0.51 ± 0.58	
Proximal–buccal	0.70 ± 0.55	

* Kruskal–Wallis test (*p* < 0.05). Post hoc Dunn test. MC: Multiple comparison.

## Data Availability

The data underlying this article are available upon reasonable request from the corresponding author.
